# *Crithmum maritimum* L.: Phytochemical Profile, Biological Activities, and Therapeutic Potential

**DOI:** 10.3390/molecules30132832

**Published:** 2025-07-01

**Authors:** Velina Dzhoglova, Stanislava Ivanova, Michaela Shishmanova-Doseva, Kremena Saracheva

**Affiliations:** 1 Department of Pharmacognosy and Pharmaceutical Chemistry, Faculty of Pharmacy, Medical University of Plovdiv, 4002 Plovdiv, Bulgaria; velina.dzhoglova@mu-plovdiv.bg; 2 Research Institute, Medical University of Plovdiv, 4002 Plovdiv, Bulgaria; mihaela.shishmanova@mu-plovdiv.bg (M.S.-D.); kremena.saracheva@mu-plovdiv.bg (K.S.); 3 Department of Pharmacology, Toxicology and Pharmacotherapy, Faculty of Pharmacy, Medical University of Plovdiv, 4002 Plovdiv, Bulgaria

**Keywords:** *Crithmum maritimum* L., herbal medicines, natural products, hepatoprotection, essential oils

## Abstract

Members of the *Apiaceae* family have been recognized since antiquity for their health-promoting properties. The halophytic species *Crithmum maritimum* L. (commonly known as sea fennel) has been used in traditional medicine since antiquity, largely due to its diverse and bioactive phytochemical composition. The plant’s complex chemical composition includes terpenoids, phenolic acids, flavonoids, tannins, dietary fibers, fatty acids, and essential vitamins. Essential oils (EOs) extracted from *C. maritimum* L. have demonstrated a wide range of biological activities, including antibacterial, antifungal, anti-inflammatory, antioxidant, and anticancer effects. Moreover, recent evidence suggests additional biofunctional roles such as cognitive enhancement and the inhibition of melanin synthesis in the skin. Extracts of the plant exhibit significant bioactivity, having shown antiparasitic, hypoglycemic, vasodilatory, and probiotic effects in preliminary studies. Despite this pharmacological potential, the number of experimental studies (particularly in vivo investigations) remains limited. The present review consolidates existing in vitro and in vivo research on *C. maritimum* L. with an analysis of 79 scientific studies aimed at elucidating its therapeutic potential and identifying future research directions necessary to support its broader application in biomedical and functional food contexts.

## 1. Introduction

*Apiaceae* is regarded as one of the largest families in the plant world. Its name originates from the name of the genus *Apium* [1]. For years, species belonging to *Apiaceae* have a strong relationship with human society. Some of the most important plants belonging to this family include carrot, parsnip, celery, coriander, fennel, cumin, anise, caraway, and others [2].

Nowadays, it is believed that this family is one of the most widespread in the plant world and consists of more than 3700 species, belonging to more than 400 genera [1,3]. Its representatives are used not only in the culinary industry but are associated with a great variety of applications in different fields, such as cosmetics, medicine, the pharmaceutical industry, and agriculture [4].

*Crithmum maritimum* L. (*Apiaceae*) is an example of a lesser studied species with significant therapeutical potential [5], and is the only member of the genus *Crithmum* [6]. *C. maritimum* L. is also called sea fennel. However, it is popular and known by other names in different countries [7]. For example, in Greece the plant is famously known as “Kritmo”, while in England it is known as Rock Samphire and St. Peter’s herb, which comes from the patron of fishermen Saint Peter, [7,8]. The word “*crithmum*” derives from the Greek language and means barley, because of their morphological similarities [7,8]. The word “*maritimum*” refers to places where the plant grows. *C. maritimum* L. naturally grows in Southern Europe and can be found mainly on coastal rocks and sandy beaches along the coast of the Black Sea and the Mediterranean Sea [7,9].

*C. maritimum* L. could also be classified as a halophyte species [8]. Halophytes, constituting only 2% of the world’s flora, and are of great interest in medicine and agriculture [10,11]. Halophyte plants have the distinctive feature of having the ability to grow in environments with a high salt content [12]. This high tolerance to the harsh conditions of their environment is a result of the development of various physiological, morphological, and biochemical alterations by these plants [12,13,14,15].

**Figure 1:**
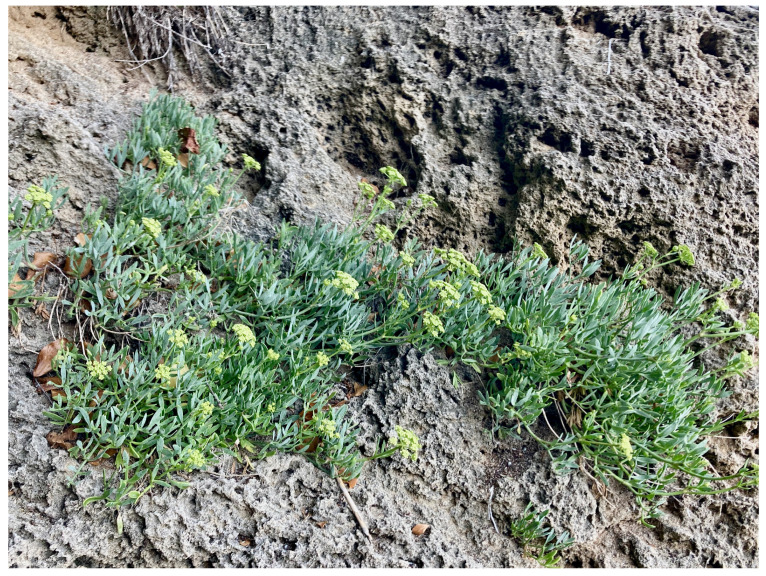
*C. maritimum* L.—wild population.

The morphological characteristics of *C. maritimum* L. (Figure 1) include a height up to 60 cm, a woody branched stem, succulent leaves, and yellow-green flowers [6]. The halophyte is a perennial plant. In Europe, it blooms between June and August [6]. Sea fennel holds significant ecological importance due to its unique adaptations and vital role in coastal ecosystems. Its extensive root system contributes to soil stabilization and erosion prevention on coastal cliffs and dunes, while also facilitating the formation and maintenance of microhabitats. Furthermore, it enhances biodiversity by providing shelter and a food source for various insects, including pollinators. Sea fennel is also considered a valuable bioindicator of coastal ecosystem health; its presence typically signifies a relatively undisturbed and natural shoreline, as it is highly sensitive to habitat degradation and environmental disturbances.

*Crithmum maritimum* L. has a long-standing tradition of culinary use, particularly in Mediterranean coastal regions. Its succulent, aromatic leaves possess a distinctive flavor profile—saline, slightly bitter, and reminiscent of fennel and celery—which makes it a valued ingredient in traditional and modern gastronomy. Sea fennel is often consumed raw in salads, pickled, or used as a seasoning for fish and seafood dishes. In recent years, its gastronomic appeal has grown due to the increasing demand for wild, foraged, and functional foods. Rich in essential oils, polyphenols, vitamins, and minerals, sea fennel not only enhances flavor but also contributes potential health benefits, aligning with current trends in health-conscious cuisine. Its culinary versatility, combined with its nutritional and organoleptic qualities, positions *C. maritimum* L. as a promising ingredient in both traditional diets and contemporary gourmet cuisine.

This plant has been recognized by humans since antiquity and has a long history of use in traditional medicine, where it was employed for its diuretic, carminative, and anthelmintic properties [7,16]. According to limited data, it has also been used in the prevention and treatment of scurvy [17]. The phytochemical composition of the essential oil and the extract from the aerial parts of this plant makes it a valuable source of innovative novel products with potential applications in the cosmetics and pharmaceutical industries. Scientific research in this direction has shown the presence of the following biological activities: anti-inflammatory, antioxidant, antibacterial, antiviral, antifungal, and anticancer activities [16,18,19].

However, most of the results, regarding its biological activity, are obtained from in vitro studies. Data from in vivo studies are very limited. Significantly more in vivo experiments need to be conducted to gain a deeper understanding of the therapeutic potential of this halophyte plant, as well as to assess its safety or toxicity, although the plant is regarded as safe and is used in the culinary traditions of many nations.

The aim of this review is to summarize the literature data on the chemical composition and in vivo and in vitro studies of *C. maritimum* L. The review represents the first comprehensive and systematic synthesis of the current scientific literature encompassing both in vitro and in vivo studies on *Crithmum maritimum* L. The work provides an integrated understanding of the plant’s phytochemical composition, biological activities, and potential applications across various domains. This review thereby addresses a significant gap in the literature and serves as a foundational reference for future research on this underexplored halophytic species.

## 2. Results

The number of studies investigating *C. maritimum* L. are limited—in total 24 studies on its biological activity were identified (10 in vitro studies on the essential oil, 14 in vitro studies on *C. maritimum* L. extracts, 1 in vivo study involving animals, and 1 study involving humans). Table 1 and Table 2 summarize the biological effects of the EO and the extracts, respectively, obtained from *C. maritimum* L. that have been proven by in vitro studies.

Only one in vivo study on animals regarding *C. maritimum* L. was found. The study was performed in France and involved male Wistar rats, separated into four groups [37]. Each group was composed of five rats: control animals, animals supplemented daily for 5 days with a water suspension of *C. maritimum* L. leaves, rats injected intraperitoneally with a single dose of 1.5 mL CCl4/kg bw, and animals given a water suspension of *C. maritimum* L. leaves daily for 5 days followed on day 5 by a single i.p. dose of 1.5 mL CCl4/kg bw [37]. According to the chemical analyses, chlorogenic acid was the major component [37]. Trans ferulic acid, cryptochlorogenic acid, meochlorogenic acid, quinic acid were also found at high levels [37]. The study suggested that the supplementation with *C. maritimum* L. to intoxicated rats restored the impaired hepatic markers and reduced induced oxidative stress, GSH, and protein carbonyl levels [37]. The plant was associated with significant hepatoprotection [37].

Marie Caucanas and colleagues studied *C. maritimum* L. stem cells for potential epidermal permeability barrier repair [38]. This is the only study on sea fennel involving humans [38]. The aim of the study was to evaluate the recovery rate of the epidermal permeability barrier function following controlled stripping and applications of skin formulations enriched with sea fennel biomass [38]. The study design included 12 healthy individuals (age < 50 years) [38]. Controlled stratum corneum stripping was used to increase transepidermal water loss, and after this there was a period of daily applications of formulations enriched with sea fennel biomass for 14 days [38]. The researchers reported that a fast recovery to lower transepidermal water loss values was obtained with each of the sea fennel formulations [38]. The process was found to be significantly faster than that in the controls [38]. No adverse reactions were recorded. The application of formulations enriched with sea fennel biomass was associated with high levels of stratum corneum moisture [38]. This study highlights the significant potential of *C. maritimum* L. biomass to be involved in some future strategies for managing the aging of the skin and its inclusion in the management of some dermatological conditions [38].

A recent study investigated the safety and biological activity of a novel dietary supplement, which is named “Antiox-Plus” [39]. *C. maritimum* L. EO was one of its components; among the other components were 10 mg of hydroxytyrosol and EOs from *Origanum vulgare* subsp. *Hirtum* and *Salvia fruticosa* [39]. Each EO was found at a quantity of 8.33 μL [39]. The study included 12 healthy volunteers and lasted 12 weeks. The participants were men and women between 26 and 52 years old [39]. The supplement was taken once a day, 15 min before the main meal [39]. The collected data from the study revealed that the 12 participants showed a reduction in blood glucose, homocysteine, and oxLDL [39]. These results indicated that the supplement had potential for use in the treatment of cardiovascular disease [39]. During the study, the intake of the supplement did not show any toxic effects [39].

## 3. Discussion

Based on the recent in vitro, and very limited, in vivo studies, some of the therapeutic potential of *C. maritimum* L. can be outlined. Different cell-based models reveal strong antimicrobial and antifungal activity, which could be used for food preservatives, antibiotics, especially against Gr (+) microorganisms, and the treatment of dermatomycosis [20,28,29,40]. The significant cytotoxic activity and anticancer potential of *C. maritimum* L. against some tumor cell lines, such as mouse lymphocytic leukemia, human myeloma, and hepatocellular carcinoma, have been described [29,34,41]. Moreover, the only in vivo study revealed the pronounced hepatoprotective effect of the crop [34]. Another interesting aspect of *C. maritimum* L. is the content of a significant number of fatty acids within the Ω-3 and Ω-6 series, suggesting beneficial effects on the prevention of coronary heart diseases [42]. The recently reported cholinesterase inhibitory activity of the essential oil and extracts of *C. maritimum* L. represents an important target in the treatment of the first stage of Alzheimer’s disease, dementia, and other neurological disorders [7,8]. Its well-established antioxidant and anti-inflammatory activity could be an Important target in different acute and chronic inflammatory diseases [4,25,43]. The potential prebiotic effect on the growth of *Lactobacillus bulgaricus* has been reported, which, taken together with the inhibitory activity on α-amylase and α-glucosidase, has the potential to be applied in the treatment of various metabolic disorders [16,30].

### 3.1. Therapeutic Potential of the EO

As can be seen from the data presented in Table 1, one of the main components in the EO of *C. maritimum* L. is β-pinene. β-Pinene, which is bicyclic monoterpene hydrocarbon that is found in the EOs of a variety of plant species, such as *Juniperus communis*, *Rosmarinus officinalis*, *Lavandula stoechas*, *Coriandrum sativum*, *Myristica fragrans*, *Cinnamomum verum*, *Achillea millefolium*, and *Piper nigrum* [44].

Most of the studies on this bioactive compound reported antibacterial activity, mainly against Gram-positive bacteria such as *S. aureus*, *S. pyogenes*, *S. pneumonia*, and *S. epidermidis*. These effects are primarily attributed to its ability to inhibit the synthesis of bacterial biofilms [45]. Studies also revealed activity against some fungi species, including *C. albicans* [46]. There are also studies showing that β-pinene also exhibits antidepressant properties by inhibiting 5-HT_1A_ receptors [47]. β-pinene has neuroprotective properties and could affect cholinergic mediation, which explains its potential in Alzheimer’s disease [48]. The gastroprotective effect of β-pinene was also observed and it was proven that β-pinene was effective against ethanol-induced gastric ulcers by increasing mucus secretion [49]. This volatile compound also has well-studied insecticidal, antioxidative, and anti-inflammatory activities [50].

γ-Terpinene is a substance that is commonly found in the EOs of various aromatic plants, for example, it can be isolated from coriander, lemon, and cumin EO [51]. γ-Terpinene is monocyclic monoterpene hydrocarbon, which occurs in high levels in the EO of sea fennel as well, according to the scientific literature [52]. An in vivo study demonstrated the anti-inflammatory properties of this volatile component, reducing the levels of IL-1β and TNF-α, and inhibiting protein accumulation in inflamed tissue [53]. The experimental model showed that γ-terpinene led to a significant decrease in the swelling caused by carrageenan [53]. A study revealed that γ-terpinene also exhibits antioxidant activity and has the ability to protect DNA from oxidation [54]. In addition, γ-terpinene also exhibited analgesic effects in neuropathic pain, related to malignant tumors [55]. The antimicrobial activity of γ-terpinene was also investigated, and it was found that the compound effectively reduced the growth of Gram-positive and Gram-negative bacterial strains, such as *S. aureus* and *E. coli*, disrupting the integrity of the bacterial cell wall and cell membrane [56]. γ-Terpinene is a bioactive plant component with rich therapeutic potential, whose fungicidal, cytotoxic, and insecticidal properties have also been studied [57,58,59].

The monoterpene limonene was found to be the major compound isolated from *C. maritimum* L. EOs from Croatia (57.5–74.2%) [24]. The authors reported great activity of the EOs against cholinesterase enzymes [24]. The reported activity could be a result of the high levels of limonene [24]. Many other studies previously associated the compound with a significant therapeutic potential for the management of Alzheimer’s disease (AD) [60]. Alzheimer’s disease is a progressive neurodegenerative disorder that primarily affects cognitive function, including memory, reasoning, and decision-making [60]. It is regarded as one of the biggest challenges for modern science [60]. The key pathological features of AD include cognitive impairment, acetylcholine (ACh) deficiency, amyloid beta plaques, and neurofibrillary tangles [60]. The treatment of AD focuses on alleviating symptoms, slowing disease progression, and improving quality of life [60]. Currently, there are no options for recovery [60]. The pharmacological treatment of AD involves cholinesterase inhibitors, NMDA receptor antagonists and combinations [60]. Nowadays, many molecules and plant extracts are studied as potential drug candidates for the management of AD [60].

Different studies evaluated the acetylcholinesterase (AChE) and butyrylcholinesterase (BChE) activity of limonene, the results revealed inhibitory activity related to these enzymes [60].

**Figure 2:**
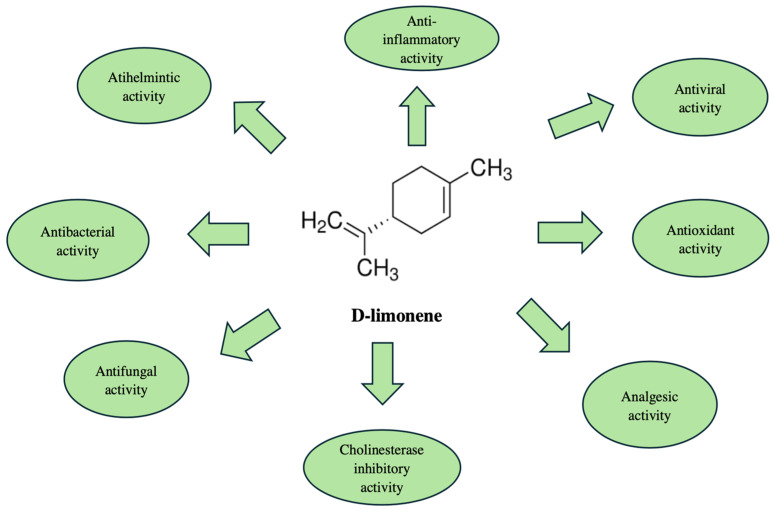
Chemical structure of D-limonene and its biological activities.

Moreover, limonene has been shown to possess notable antibacterial properties, with recent studies shedding light on its potential mechanism of action [61,62]. In 2019 Yingjie Han, Zhichang Sun and Wenxue Chen reported that limonene can compromise the integrity of the *Listeria monocytogenes* cell membrane, leading to structural damage and impaired cellular function [62]. In 2021, Gupta, Jeyakumar, and Lawrence published a study investigating the antibacterial properties of limonene against multidrug-resistant pathogens. Their findings revealed that limonene induces membrane disruption, resulting in cellular leakage and subsequent death of *Escherichia coli* cells. The release of intracellular components, including proteins, lipids, and nucleic acids, provided clear evidence of membrane damage and compromised cell permeability [61]. Figure 2 illustrates the main biological effects of D-limonene and its chemical structure.

**Figure 3:**
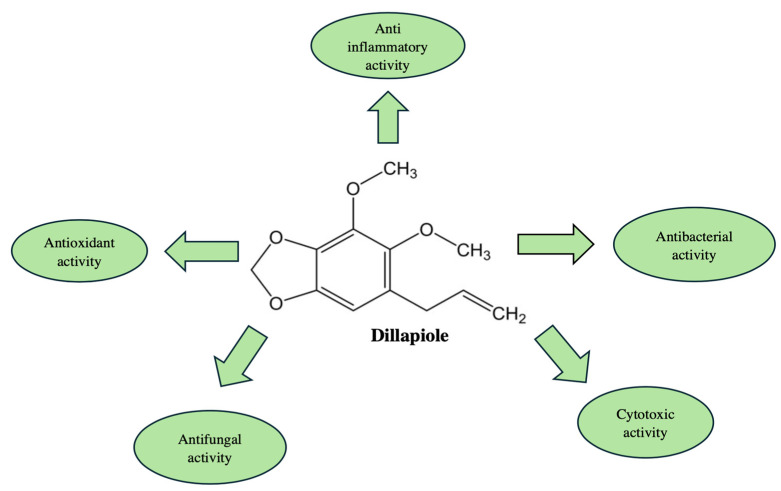
Chemical structure of dillapiole and its biological activities.

*C. maritimum* L. contains significant quantities of dillapiole, which is a representative of phenylpropane derivatives [63]. *Piper aduncum*, *Anethum graveolens*, and *Perideridia gairdneri* are medicinal plants, whose EOs also contain this phenylpropanoid [64,65,66]. The latest research demonstrated the anticholinesterase activity of dillapiole at high doses in the larvae of *Amblyomma sculptum* [67]. This result suggested the potential formulation of effective acaricidal products containing dillapiole [67]. Another study reveals for the first time that dillapiole has a wide range of cytotoxic effects and is effective against different types of tumor cells [68]. In this study, dillapiole-induced cell apoptosis is carried out by several mechanisms, including through the generation of reactive oxygen species (ROS) and as a result of disruption of mitochondrial functions [68]. The anti-inflammatory activity of dillapiole has also been demonstrated through an in vivo study involving rats [69]. The results of this study show that dillapiole reduced tissue edema, making the substance a potential source of safer alternatives to NSAIDs [69]. The effectiveness of dillapiole in diabetic nephropathy was also investigated [70]. The experimental model showed that dillapiole reduced the levels of important indicators in blood plasma such as glucose, total cholesterol, triglycerides, LDL, and VLDL [70]. It is assumed that these results were due to the ability of dillapiole to activate PPARγ [70]. Figure 3 represents the main biological effects of dillapiole and its chemical structure.

### 3.2. Therapeutic Potential of the Extracts

As demonstrated in Table 1 and Table 2, sea fennel exhibits some differences in its biological composition, as well as in its therapeutic potential, depending on extraction technic.

Sea fennel’s extracts are a rich source of hydrophilic (polyphenols, vitamin C) and lipophilic (carotenoids, essential oils, fatty acids) bioactive compounds. According to some researchers, *C. maritimum* L. is among the highest phenolic-containing species in the *Apiaceae* family [43,71]. The content of antioxidant enzymes, phenolic compounds, carotenoids, and chlorophylls could be responsible for the potential antioxidant activity of the crop. In humans, they exhibit important beneficial health effects, mainly by preventing oxidative stress. Therefore, they are widely considered to be useful in the prevention of diabetes, cancer, cardiovascular diseases and neurological disorders [72]. According to Zengin et al., the effects of phenolic compounds on free radicals appear to be a primary factor in the antioxidant effect of the extracts [73].

Polyphenols are a large group of plant metabolites displaying key functions [43,71]. Many studies revealed a high content of phenolic acids, namely chlorogenic ones, in *C. maritimum* L. extracts [43,71]. As it is well known, chlorogenic acids have a number of biological activities, including antioxidant, antimicrobial, anti-inflammatory, anti-carcinogenic, etc. These statements are also supported by Santana-Gàlvez et al. who have proposed that the antioxidant properties of the crop are namely due to the chlorogenic acids, which significantly increase ROS scavenging activities [24,74]. Previous studies have considered the involvement of other compounds as flavonoids and their derivatives to also play a crucial role in the antioxidant effect of the extract [74,75].

According to Ailén et al., the high chlorogenic acids content of both sea fennel extracts—aqueous and ethanolic—determines its anti-inflammatory activity, being a result of a reduced release of TNF-α and increased IL-10 secretion in macrophages [33]. In addition to what has been described so far, the content of chlorogenic acids also determines the antimicrobial activity of the plant against a wide variety of bacteria, including *S. aureus*, *S. pneumoniae*, *B. subtilis*, *B. cereus*, *E. coli*, *E. faecalis*, and *S. typhimurium* [28]. In addition, *C. maritimum* L. extract showed a significant content of rutin and quercetin derivatives, which proved to be efficient inhibitors of the growth of *E. coli*, *S. aureus*, *E. faecalis*, and *P. aeruginosa* [28].

Chlorogenic acids have been revealed to have a significant therapeutic impact in influencing lipid and glucose metabolism [76]. The antidiabetic properties of this compound have been linked to reduced glucose absorption in the small intestine and decreased glucose release due to the inhibited expression of hepatic glucose-6-phosphatase [76]. Therefore, this limits the release of blood glucose in general circulation, followed by the lowering of insulin release [76]. These effects are accompanied by less fatty deposition, increased burning of excessive adipose tissue, and weight reduction [76]. Moreover, recent research described the potential hypoglycemic capacity of aqueous extracts from sea fennel with a high content of polyphenols, which could be responsible for the dipeptidyl peptidase IV-inhibiting activity [77].

Potential prebiotic effects on the growth of *L. bulgaricus* have been reported by Correia et al. [30]. They found faster growth of *L. bulgaricus* in the presence of sea fennel leaves extract, suggesting a possible prebiotic effect due to its high content of phenolic acids, flavanols, and anthocyanins [30].

An additional therapeutic potential of *C. maritimum* L. extract is its hypotensive effect, which is due to its vasodilatory activity [24]. This effect is associated with the presence of chlorogenic acid and other phenolic compounds and has been demonstrated in experimental and human studies [24,78,79]. The authors outlined several mechanisms which could be responsible for the observed effect, including antioxidant activity and enhanced production of some vasodilating agents, such as nitric oxide and endothelium-derived hyperpolarizing factor [24,78]. Another interesting aspect of *C. maritimum* L. is the content of a significant amount of fatty acids within the Ω-3 and Ω-6 series suggesting beneficial effects on the prevention of coronary heart diseases [42].

A recent study demonstrated that sea fennels’ active compounds, including flavonoids and peptides, could enhance the collagen synthesis of fibroblasts, especially type I collagen [35]. This promotes epidermal regeneration, fostering the production of new skin tissue, skin hydration, and elasticity [35].

Recent investigations by Pereira et al. [36] revealed that two of the components of *C. maritimum* L. extract are falcarinol and falcarindiol, representing aliphatic C_17_-polyacetylenes of the falcarinol type [36]. These molecules are namely responsible for the anti-trypanosomal activity of the extract against some *T. species* such as *T. brucei* and *T. cruzi* [36]. Although the exact mechanisms of action are still not well elucidated, it has been demonstrated that their alkylating properties are responsible not only for their antiparasitic effect but for many other bioactivities, including antibacterial, antifungal, anti-inflammatory, neurotoxic, cytotoxic, and allergenic effects [36,80]. Previous studies by Meot-Duros et al. found that one of the already mentioned components isolated from sea fennel leaves, falcarindiol, has a strong and wide spectrum of antimicrobial activity against *S. arizonae*, *E. carotovora*, *P. fluorescens*, *P. aeruginosa*, *P. marginalis*, *B. cereus, and M. luteus* with minimum inhibitory concentration values ranging from 1 to 100 μg ml^−1^ [71]. Furthermore, the antifungal activity of sea fennel extracts from aerial parts has been reported in in vitro studies against the genus *Candida*, particularly *C. albicans*, with a minimum inhibitory concentration (MIC) of 75.0 µg/mL [30,32].

Gnocchi et al. described the role of the sea fennel ethyl acetate extract, rich in flavonoids, falcarindiol, carotenoids, etc., in inhibiting the growth of hepatocellular carcinoma cells [81]. The authors have demonstrated that the reduction in tumor growth is a result of activated aerobic oxidative metabolism through decreased lactic fermentation and activated oxidative phosphorylation [81]. All these data suggest that the antitumor effect of the crop is due to the modulation of cellular metabolism [81]. Other reviews also confirmed the presence of in vitro cytotoxic activity of *C. maritimum* L. extract against some other tumor cell lines, such as mouse lymphocytic leukemia and human myeloma, without cytotoxic effects against normal human intestinal cells [30].

## 4. Materials and Methods

The search strategy was directed towards studies relating to bioactive compounds isolated from *C. maritimum* L. and their biological activities. An expanded search for articles without language restrictions was conducted on the following databases: Google Scholar, PubMed, Scopus, and Web of Science. In the search process, the following key words were used: “*Crithmum maritimum* L.”, “*crithmum*”, “sea fenel”, “Kritmo”, “Rock Samphire”, “in vivo studies”, “in vitro studies”, “clinical trials”, and “bioactive compounds”.

## 5. Conclusions

*Crithmum maritimum* L., commonly known as sea fennel, has emerged as a plant of considerable interest due to its diverse phytochemical composition and multifaceted potential across various sectors, including medicine, cosmetics, and gastronomy. Its richness in dietary fiber, essential nutrients, trace minerals, and antioxidant compounds supports its classification as a functional food with potential therapeutic value. These attributes have led to increased scientific investigation into its biological activities, particularly its antioxidant, anti-inflammatory, antimicrobial, and cytoprotective properties.

Despite these promising findings, substantial gaps remain in our understanding of its mechanisms of action, pharmacodynamics, and bioavailability. To date, most studies have been limited to in vitro assays, with a scarcity of in vivo and clinical investigations. Therefore, future research should aim to elucidate the molecular pathways influenced by *C. maritimum* L. extracts, assess their efficacy and safety in animal models and human populations, and evaluate possible toxicological risks. Addressing these gaps will be critical to fully harnessing the plant’s potential and facilitating its safe integration into pharmacological, nutraceutical, and commercial applications.

## Figures and Tables

**Table 1:** *C. maritimum* L. essential oils—in vitro studies.

Study Design	Country	Main Bioactive Compounds	Main Findings	Ref.
The antibacterial activity was evaluated by a paper-disk diffusion technique. The antibacterial activity study included eight bacteria species including *S. aureus*. Different studies evaluated the acetylcholinesterase, *B. subtilis*, *B. cereus*, *P. aeruginosa*, *P. mirabilis*, *E. coli*, and *S. typhi*. The concentrations of *C. maritimum* L. EO used were from 30 to 1.87 mg/mL. Positive controls—gentamycin and tetracycline.	Turkey	The major identified compounds were β-phellandrene (30%) and thymol methyl ether (25%). Other compounds, which were found, were γ-terpinene (24%) and dillapiole (21%).	The EO from *C. maritimum* L. exerted antibacterial activity, especially against Gram-positive bacteria. The most sensitive strain was *B. cereus*. *P. mirabilis* had no sensitivity to the EO.	[20]
The antimicrobial activity of the *C. maritimum* L. EO was evaluated by the disc diffusion method. The concentration of the tested EO was 5%. The EO was examined against methicillin-sensitive *S. aureus* and methicillin-resistant *S. aureus*. As positive controls were used penicillin, enrofloxacin, gentamicin sulfate, tetracycline hydrochloride, and cefaclor.	Greece	The main substances in the EO were 1,8-cineol (39.70%) and β-phellandrene (28.01%).	The EO did not demonstrate sufficient antimicrobial activity.	[21]
The antifungal activity was elevated against *C. albicans*, *C. guillermondii*, *C. neoformans*, *E. floccosum*, *T. mentagrophytes*, *M. canis*, and *M. gypseum*. The method used was macrodilution broth. Concentrations of 0.02–20 µL/mL of the EO were used. Fluconazole was used as a positive control.	Portugal	The major compound was dillapiole (64.2%). p-Cymene (11.8%), γ-terpinene (40.1%), and thymol methyl ether (23%) were also found in the EO.	The EO from *C. maritimum* L. has antifungal properties and it has the potential to be applied as a treatment of dermatophytosis.	[22]
The antioxidant activity was evaluated, using the 2,2’-azinobis-3-ethylbenzthiazoline-6-sulphonic acid (ABTS) radical-scavenging assay. The amount of EO used was 20 µL. The EO was mixed with ABTS. Ascorbic acid was used as a positive control.	Tunisia	The dominant compound was dillapiole (2.39–41.35%). Other components found in the EO were thymol methyl ether (20.13–34.75%), p-cymene (4.83–22.08%), and γ-terpinene (22.54–43.29%).	The tested EO from *C. maritimum* L. demonstrated antioxidant properties.	[23]
The antioxidant potential of *C. maritimum* L. EO was estimated as having ferric reducing/antioxidant power (FRAP), using DPPH assay. The concentration used was 0.1 mg/mL. The results were obtained after measurement with a spectrophotometer.	Croatia	Limonene was the major compound in the EO (57.5–74.2%). Other compounds at high levels were **γ**-terpinene (13.8–4.6%) and sabinene (13.4–8.1%).	The study revealed a low antioxidant capacity of the isolated EO.	[24]
The study investigated anti-inflammatory activity and involved cell line RAW 264.7 (ATCC TIB-71)—a mouse leukemic macrophage cell culture. This activity was estimated using the levels of nitrite production. For the cell stimulation, lipopolysaccharide was used. The concentrations of the applied EO from *C. maritimum* L. were 50–3.125 μg/mL. The results were obtained using an automatic microplate reader, measuring the absorbance.	Portugal	The main substances were γ-terpinene (33.6%), sabinene (32.0%), and thymol methyl ether (15.7%).	The EO demonstrated an anti-inflammatory activity and no toxicity.	[25]
The acetylcholinesterase inhibitory activity was evaluated by the presence of a yellow color by 2-nitrobenzoic acid and thiocholine, produced by acetylcholinesterase. The inhibitory activity was assessed based on the intensity of the yellow color obtained. The activity of the EO was compared to donepezil and tropolone. After incubation, the results from the study were obtained after absorbance measurement and calculations, which demonstrated acetylcholinesterase-inhibitory activity.	Libya	The major compounds found were thymyl methyl ether (56.86%), and γ-terpinene (16.17%).	The EO demonstrated a acetylcholinesterase-inhibitory effect. This finding revealed future application of the EO as an enhancer of cholinergic neurotransmission, improving the symptoms of Alzheimer’s disease.	[26]
The cholinesterase-inhibitory activity of *C. maritimum* L. EO was estimated, using a modified Ellman method. First, 10 µL of the sample was applied. Ethanol was used as a negative control. Non-enzymatic hydrolysis was also monitored. A spectrophotometer was used for all measurements.	Croatia	Limonene (57.5–74.2%) was the major compound in the EO. Other constituents in the EO were γ-terpinene (4.6–13.8%), β-pinene (0.1–4.9%), thymol methyl ether (0.4–0.2%), and terpinen-4-ol (6.9–2.0%).	The study showed that *C. maritimum* L. EO possessed cholinesterase inhibitory activity.	[24]
The estimation of the tyrosinase-inhibitory activity was performed using spectrophotometry. A mixture of a solution of tyrosine and a methanol solution of the enzyme inhibitor—arbutin—was prepared. After the addition of mushroom tyrosinase solution, the tyrosine was oxidized. The EO from *C. maritimum* L. was also prepared and tested. Methanol solution was used as a control.	Libya	The main constituents were thymyl methyl ether (56.86%), and γ-terpinene (16.17%).	The EO exerted tyrosinase-inhibitory activity. Consequently, the EO could be used in skincare, targeting melanin synthesis for the purpose of reducing hyperpigmentation.	[26]
A study of the anticancer activity of *C. maritimum* L. EO was conducted using the following cell lines: HEK293-T (human embryonic kidney), RKO (colorectal cancer), and MCF7 (breast cancer). The applied concentrations of the EO were 1 μL/mL to 10^−5^ μL/mL. Cell viability and growth inhibition were observed. Doxorubicin hydrochloride was used as a reference.	Portugal	The major bioactive compounds were γ-terpinene, thymol methyl ether, o-cymene, and β-phellandrene. Each of these compounds was found to be more than 10% of the EO.	The isolated EO did not demonstrate significant cytotoxic activity in the tested cell lines.	[27]

**Table 2:** *C. maritimum* L. extracts—in vitro studies.

Study Design	Country	Main Bioactive Compounds	Main Findings	Ref.
The study of the antibacterial activity of the *C. maritimum* L. extract involved five bacteria species: *S. aureus, E. faecalis, E. coli, E. aerogenes, and S. enterica* ser. *typhimurium.* The extract was diluted with sterile water to the concentration of 0.05 mg/mL. Then 100 µL of the extract was added to different cultivated bacteria strains. The negative control was water. The antibacterial activity was evaluated by measuring the final optical density with a microplate reader.	France	The main compound in the extract was chlorogenic acid. Quinic acid, neochlorogenic acid, and trans ferulic acid were the other compounds found at high levels.	Hydro-ethanolic extract of *C. maritimum* L. leaves exerted strong antibacterial activity against Gram-positive and Gram-negative bacteria.	[28]
Purified falcarindiol, obtained from *C. maritimum* L. extract, was tested against Gram-positive and Gram-negative bacteria: *M. luteus*, *B. cereus*, *S. enterica subsp. arizonae*, *P. fluorescens*, *P. marginalis*, *E. coli*, and *E. carotovara subsp. carotovora*. The compound was studied for antifungal activity as well—against *C. albicans*. The applied concentrations of falcarindiol were 10, 20, and 50 µg/mL. Streptomycin and penicillin G were used as positive controls.	France	The test was performed using one of the main compounds from *C. maritimum* L. extract: falcarindiol.	It was found that falcarindiol affected the microorganisms differently. *M. luteus* and *B. cereus* were the most affected.	[29]
*C. maritimum* L. extract was studied for its activity against *E. coli*, *K. pneumonia*, *S. aureus*, and *S. epidermidis*. The concentrations of the test solutions were 2.34 to 600 µg/mL. Cefotaxime was used as a positive control. Minimal bactericidal concentrations and minimal inhibitory concentrations were measured.	Portugal	The study assessed the presence of minerals, Ω-6, and Ω-3 fatty acids, and polyphenols.	The extract revealed antibacterial activity, especially against the tested Gram-positive microorganisms: *S. aureus* and *S. epidermidis*.	[30]
The antioxidant activity of *C. maritimum* L. extract was evaluated using a purple-colored methanol solution of 2,2-diphenylpicrylhydrazyl (DPPH). To the 5 mL of a 0.004% methanol solution of DPPH was added 50 µL of the extract. Stable antioxidants were used as references. Hydrogen atom or electron donation abilities were assessed measuring the absorbance of the extract and the standards.	Algeria	Phenolic compounds were present at high levels in the compound, such as chlorogenic acid, 3-caffeoylquinic acid, 5-caffeoylquinic acid, and 4-caffeoylquinic acid.	Hydro-methanolic extract of aerial parts of the plant revealed antioxidant activity, and it was found that hydroxycinnamic acid derivatives were responsible for this activity.	[31]
The antioxidant activity of a methanolic extract of *C. maritimum* L. was assessed using the DPPH test. The prepared concentrations were 0.5, 1, 2, 5, and 10 mg/mL. Ascorbic acid, butylated hydroxytoluene, and quercitine were used as reference solutions. A solution of DPPH was prepared and mixed with the tested and the referent solutions.	Tunisia	The study presented the total polyphenol content, flavonoid content, and tannin content.	*C. maritimum* L. extract demonstrated antioxidant activity, due to the presence of phenolic compounds.	[32]
The anti-inflammatory activity of an aqueous extract from *C. maritimum* L. was tested using THP-1 cells. The tested cells were stimulated with lipopolysaccharide and treated with free aqueous extract at concentrations of 640 and 320 μg/mL. The aqueous extract was included in liposomes at concentrations of 1640 and 820 μg/mL. The levels of IL-10 and TNF-α were used to estimate the anti-inflammatory activity.	Spain	Chlorogenic acid.	Both extracts from *C. maritimum* L. demonstrated the induced release of IL-10 and reduced release of TNF-α, which is evidence for anti-inflammatory activity.	[33]
The study of anticancer activity was performed using two cell lines: Huh7 and HepG2. Ethyl acetate extracts of *C. maritimum* L. at concentrations of 2, 1, 0.5, 0.25 μM were used as test solutions. The proliferation of the cells was assessed with crystal violet and then the absorbance was measured by a plate reader.	Italy	No data provided	The plant extract showed a reduction in the growth of hepatocellular carcinoma cells. It also enhanced apoptosis and decreased cell cycle progression.	[34]
A study regarding the cytotoxic activity of falcarindiol, obtained from *C. maritimum* L. extract, was also performed. Small intestine cell lines from rats (IEC-6 cells) were used for the assay. The applied concentrations of the compound were 0.2 to 20 µM. The results were obtained after measuring the absorbance.	France	The assay was carried out using one of the main compounds from *C. maritimum* L. extract: falcarindiol.	Falcarindiol, isolated from *C. maritimum* L., exerted cytotoxic activity and induced a decrease in cell viability, but only at the highest concentration.	[29]
The cytotoxic activity of *C. maritimum* L. extract was estimated, using Caco-2 intestinal epithelial cell lines. 1, 2, 3, 4, 5% were the applied concentrations of the plant extract.	Portugal	The extract was rich in minerals, Ω-6, and Ω-3 fatty acids, and polyphenols.	The extract showed low toxicity concerning human intestinal epithelial Caco-2 model cells.	[30]
The assay was conducted using ethanol extracts from *C. maritimum* L. at a concentration of 30%. The cell line cultures cinsisted of keratinocytes and fibroblasts. The cell damage was induced by UVA radiation. The regenerative properties of the cells were evaluated by observing the histological morphology, loricrin content, collagen content, and collagen fiber content.	China	Chlorogenic acid	The study revealed better epidermal thickness and a notable increase in collagen fibers, collagen I, and loricrin.	[35]
Antiparasitic activity of hydro-ethanolic extracts from *C. maritimum* L. aerial parts was assessed on LLC-MK2 and U2OS cell lines. The cells were infected with *Trypanosoma cruzi*. The extract was diluted with DMSO in concentrations of 5, 10, and 20 mg/mL. Benznidazole was used as a positive control.	Portugal	Falcarindiol	The extract exerted antiparasitic activity against *Trypanosoma cruzi*. During the study, no cell line toxicity was demonstrated.	[36]
The hypoglycemic activity of the aqueous extracts from the leaves and flowers of *C. maritimum* L. was evaluated by measuring the inhibition of carbohydrate-hydrolyzing enzymes: α-amylase and α-glucosidase. In the α-amylase-inhibitory study, a starch solution with α-amylase was added to the extract in concentrations from 25 to 1000 μg/mL. For the α-glucosidase assay, the enzyme was added to maltose and o-dianisidine solutions and a peroxidase/glucose oxidase system color reagent. Then it was mixed with the extract of the plant at the same concentrations. Acarbose was used as a positive control.	Italy	The main compound in the extract was chlorogenic acid. Other identified compounds include cryptochlorogenic acid, neochlorogenic acid, rutin, and quercetin.	The plant extract possessed promising inhibitory activity against α-amylase and α-glucosidase and has the potential to be applied in the treatment of metabolic diseases.	[16]
The vasodilatory activity of *C. maritimum* L. extract was tested by estimating the isometric force of the isolated rat aortic rings. The rings were processed with ethanol extract in concentrations of 0.5‰ to 6‰ in organ baths. The relaxation of the vascular rings was presented as a percentage decrease in the stimulated vasoconstriction by noradrenaline.	Croatia	The study investigated the total phenol content, and the presence of chlorogenic acid in the plant extract is also reported.	*C. maritimum* L. extract revealed vasodilatatory activity, due to the presence of chlorogenic acid.	[24]
The prebiotic effect of *C. maritimum* L. extract was assessed, regarding *Lactobacillus bulgaricus*. The bacterial growth was assessed during aerobic conditions. The applied concentration of the extract was 0.1%.	Portugal	The extract was rich in minerals, Ω-6, and Ω-3 fatty acids, and polyphenols.	The results suggested accelerated growth of *Lactobacillus bulgaricus* after the exposure to *C. maritimum* L. extract.	[30]

## Data Availability

No new data were created or analyzed in this study. Data sharing is not applicable to this article.
